# The changing patterns of comorbidities associated with human immunodeficiency virus infection, a longitudinal retrospective cohort study of Medicare patients

**DOI:** 10.1097/MD.0000000000025428

**Published:** 2021-04-23

**Authors:** Nick D. Williams, Vojtech Huser, Frank Rhame, Craig S. Mayer, Kin Wah Fung

**Affiliations:** a The Lister Hill National Center for Biomedical Communications at the National Library of Medicine, National Institutes of Health in the United States, Bethesda, Maryland; b Division of Infectious Diseases and International Medicine at the University of Minnesota School of Medicine, Minnesota.

**Keywords:** acquired immune deficiency syndrome, comorbidities, human immunodeficiency virus, Medicare

## Abstract

The objective of this paper is to determine the temporal trend of the association of 66 comorbidities with human immunodeficiency virus (HIV) infection status among Medicare beneficiaries from 2000 through 2016.

We harvested patient level encounter claims from a 17-year long 100% sample of Medicare records. We used the chronic conditions warehouse comorbidity flags to determine HIV infection status and presence of comorbidities. We prepared 1 data set per year for analysis. Our 17 study data sets are retrospective annualized patient level case histories where the comorbidity status reflects if the patient has ever met the comorbidity case definition from the start of the study to the analysis year.

We implemented one logistic binary regression model per study year to discover the maximum likelihood estimate (MLE) of a comorbidity belonging to our binary classes of HIV+ or HIV– study populations. We report MLE and odds ratios by comorbidity and year.

Of the 66 assessed comorbidities, 35 remained associated with HIV– across all model years, 19 remained associated with HIV+ across all model years. Three comorbidities changed association from HIV+ to HIV– and 9 comorbidities changed association from HIV– to HIV+.

The prevalence of comorbidities associated with HIV infection changed over time due to clinical, social, and epidemiological reasons. Comorbidity surveillance can provide important insights into the understanding and management of HIV infection and its consequences.

## 1 Introduction

The United States (US) has seen the epidemiology of human immunodeficiency virus/acquired immune deficiency syndrome (HIV/AIDS) shift drastically since the identification of the epidemic in 1981. Originally reported in the US as a rare disease of very few urban, white homosexual men in Los Angeles, HIV has become a global pandemic infecting tens of millions and has contributed to a substantial mortality burden inside and outside the US.^[1–3]^ Without a cure or vaccine, HIV will likely remain a major healthcare concern for generations to come, necessitating the re-evaluation of the clinical vulnerabilities of HIV positive patients.

The U.S. Centers for Disease Control and Prevention (CDC) reports around 1.3 million patients diagnosed with AIDS since the beginning of the epidemic through 2017 in the US.^[3]^ Incidence of progression to AIDS and related mortality are often used as standard metrics for evaluating HIV patient outcomes. In view of the decreasing AIDS incidence and mortality, monitoring comorbidity among HIV positive patients could become an important effort in understanding and evaluating the clinical management options and outcomes for HIV disease itself.^[3,4]^

The increase in age at diagnosis in the US, the proliferation of infections among heterosexuals in urban and rural settings, and the higher prevalence of HIV among African Americans demonstrate that the HIV epidemic in the US affects different sub-populations at different points in time.^[3,5]^ Patients who are already sick with common health conditions like diabetes, obesity, and other poverty-related conditions when they contract HIV will still face those pre-existing conditions post HIV diagnosis, further complicating HIV management. Comorbidities such as drug and substance abuse can have complex relationship among themselves and with HIV transmission and progression.^[6–9]^ Injection drug use facilitates transmission of HIV and other infections that often co-occur with HIV. HIV-related neuropathological disorders such as HIV-associated dementia (HAD) and HIV-associated neurocognitive disorders (HAND) are more common in drug abusers than in other risk groups.^[10]^ The chronic inflammatory response to infection and toxicities from anti-retroviral therapy may also be implicated in comorbidities that HIV positive patients experience, in addition to the direct effects of HIV infection.^[11,12]^

In this study, we examined 66 comorbidities to assess the degree to which they were associated with HIV positive patients, and how that association changed over time. Our primary source of data was Medicare claims data from 1999 to 2016. Our sample is larger than most published studies, covering about a quarter of known patients living with HIV in the US. Our study contributes a longitudinal, population level perspective over a period that witnessed significant changes in the demographics and treatment options of HIV infection.

## 2 Methods

### 2.1 Study population, HIV status, and comorbidity definitions

Our data was sourced from the Virtual Research Data Center's (VRDC) chronic conditions warehouse (CCW).^[13]^ We acquired access to a 100% sample of Medicare encounter level claims data spanning 1999 to 2016. Our first observation year was 2000, and our final year was 2016. Not all cases entered the study on the same start day or at the same age, and cases only left on death. Cases and controls could enroll in Medicare and be included in the study at any time over the study period.

HIV positive cases were defined using the CCW HIV/AIDS case definition. To be HIV positive Medicare beneficiaries needed 1 inpatient or 2 outpatient claims within 2 years demonstrating explicit The International Classification of Diseases, Ninth Revision, Clinical Modification (ICD-9-CM), The International Classification of Diseases, Tenth Revision, Clinical Modification (ICD-10-CM), Medicare Severity-Diagnosis Related Group (MS DRG) or CMS hierarchical condition category (HCC) codes for HIV infection. We considered this case definition as conservative and had a low likelihood of false positivity but could potentially under-identify cases such as patients who did not bill HIV-related services through Medicare. Therefore, we supplemented the Medicare HIV flag with the HIV flag from Medicaid data, even though morbidity data only came from Medicare. There was a high degree of overlap of HIV positive patients between the 2 sources. The vast majority (over 99.9%) of the HIV positive Medicaid patients were dual-enrolled to both Medicare and Medicaid over the study period. The first date of an HIV-related code might only reflect the first service claim and not the start of infection. Therefore, for all patients who were ever flagged as HIV positive, we assumed that they had been positive from the beginning of their observation period.

We defined HIV negative controls as individuals who were never flagged for HIV positivity at any time in the whole study period. This was necessary to avoid erroneously recruiting HIV positive cases as controls since we did not have a definite start date of HIV infection. Controls were enrolled on an annual basis from the Medicare population, which was the sole source of comorbidity data. Controls were matched on case sex, race/ethnicity, 5-year age groups, and the first 3 digits of the home zip-code (our strata). Five controls were enrolled for each case using simple random sampling within the matching strata. Controls were re-assigned every year because of the attrition of both cases and controls through mortality and dis-enrollment from Medicare.

Similar to HIV status, comorbidity identification also relied on the CCW flags. The full list of case definitions is available as supplementary material. A comorbidity was identified in a patient if it was ever flagged in any year prior to and including the observation year. This cumulative approach was adopted because most of the comorbidities were chronic conditions which should persist once diagnosed, but not all of them would necessarily appear on a claim within a given year.

### 2.2 Statistical method

This study focused on the changing trend of comorbidities that were associated with HIV infection over the 17 years observation period. To quantify the association of comorbidities, we created a logistic regression model for each study year, using all comorbidities as covariates and HIV status as the variable of interest. This method is commonly employed among logistic regression association studies.^[14–17]^ It is important not to confuse this with a cause-and-outcome study, which typically uses exposure as covariates and disease as the outcome variable of interest. What we are examining is the association (not causality) between comorbidities and HIV infection. By including all comorbidities in one annual regression model (instead of separate models for each morbidity), we can adjust for the influence of multiple comorbidities co-occurring in the same patient at the same time.

From each year's regression model, we obtained the maximum likelihood estimate (MLE) for each comorbidity as an indicator of the level of association with HIV infection. Simply put, the MLE is the weight assigned to each comorbidity in the regression model after the model has been optimized to yield the highest probability (maximum likelihood) that the observed data can be explained by the model. The higher the MLE, the more important a comorbidity is in explaining the difference between HIV patients and controls. A positive MLE generally indicates that a comorbidity is more likely to be seen in HIV infected patients and a negative MLE indicates that it happens more in the controls. The magnitude of association is reflected in the absolute value of the MLE. Annual case counts, MLE, and standard errors are available in supplementary materials for all 66 comorbidities.

Ours was an unmatched analysis, meaning that our logistic regression models did not know which patient was matched to which control. It was a pragmatic choice since the sheer number of strata in our study would make matched analysis prohibitively expensive to compute. Unmatched regression has been compared with matched regression and found to be acceptable and may even have some advantages (e.g., improved causality attribution) in certain circumstances.^[18–20]^

## 3 Results

### 3.1 Study population and controls

Table 1 details a total of 411,904 distinct HIV positive patients with 4,054,116 patient-years of observation. There were 20,242,935 HIV negative controls. Yearly under matched rate (<5 controls) ranged from 0.08% to 0.26%. Overall, 39% of HIV positive patients died in the study period. The yearly mortality rate ranged from 3.63% to 4.59%. Survivors had an average of 9 observation years, and mortality cases had an average of 7 observation years. Compared with the estimated HIV incidence and prevalence in the US from the CDC statistics for 2016, our cohort represented about 25.7% of patients with known HIV infection in the US.^[21]^ We had more women (31% vs 23%), white (48% vs 30%) patients, and fewer Hispanics (7% vs 21%). Our patients tended to be older, with 63% (vs 32%) over 55 years old.

**Table 1:** Case demography by analysis year.

Study year	Observed cases	Mortality	Median age at death	Female	White	Black	Other	Asian	Hispanic	American Native	Median age	13–24^∗∗^	25–34	35–44	45–54	55+
2000	182,089	6816 (3.74%)	46	54,530 (29.95%)	97,120 (53.34%)	65,530 (35.99%)	2561 (1.14%)	1495 (0.82%)	13,062 (7.17%)	910 (0.50%)	47	1,599 (0.88%)	17,837 (09.80%)	56,630 (31.10%)	41,047 (22.54%)	64,957 (35.67%)
2001	192,648	7174 (3.72%)	47	58,335 (30.28%)	102,476 (53.19%)	69,867 (36.27%)	2585 (1.34%)	1634 (0.85%)	13,665 (7.09%)	957 (0.50%)	48	1632 (0.85%)	16,220 (08.42%)	57,743 (29.97%)	46,194 (23.98%)	70,842 (36.77%)
2002	203,145	7384 (3.63%)	48	62,227 (30.63%)	107,786 (53.06%)	73,991 (36.42%)	2787 (1.37%)	1775 (0.87%)	14,260 (7.02%)	992 (0.49%)	49	1726 (0.85%)	14,893 (07.33%)	57,879 (28.49%)	51,282 (25.24%)	77,343 (38.07%)
2003	213,694	7757 (3.63%)	49	66,078 (30.92%)	112,952 (52.86%)	78,305 (36.64%)	2976 (1.39%)	1906 (0.89%)	14,865 (6.96%)	1058 (0.50%)	50	1814 (0.85%)	14,078 (06.59%)	57,491 (26.90%)	56,098 (26.25%)	84,199 (39.40%)
2004	224,276	8234 (3.67%)	51	69,849 (31.14%)	117,967 (52.60%)	82,735 (36.89%)	3166 (1.41%)	2039 (0.91%)	15,449 (6.89%)	1106 (0.49%)	50	1812 (0.81%)	13,676 (06.10%)	56,729 (25.29%)	60,991 (27.19%)	91,053 (40.60%)
2005	231,456	8905 (3.85%)	52	72,218 (31.20%)	121,393 (52.45%)	85,491 (36.94%)	3271 (1.41%)	2153 (0.93%)	15,838 (6.84%)	1165 (0.50%)	51	1610 (0.70%)	12,622 (05.45%)	54,491 (23.54%)	65,048 (28.11%)	97,675 (42.20%)
2006	241,166	9406 (3.90%)	54	75,932 (31.49%)	125,345 (51.97%)	90,185 (37.40%)	3503 (1.45%)	2264 (0.94%)	16,456 (6.82%)	1230 (0.51%)	52	1653 (0.69%)	12,486 (05.18%)	52,579 (21.80%)	69,994 (29.02%)	104,441 (43.31%)
2007	246,879	9601 (3.89%)	56	78,111 (31.64%)	127,783 (51.76%)	92,887 (37.62%)	3638 (1.47%)	2353 (0.95%)	16,797 (6.80%)	1278 (0.52%)	53	1505 (0.61%)	12,054 (04.88%)	49,152 (19.91%)	73,236 (29.66%)	110,921 (44.93%)
2008	251,049	10,060 (4.01%)	59	79,514 (31.67%)	129,161 (51.45%)	95,232 (37.93%)	3773 (1.50%)	2415 (0.96%)	17,086 (6.81%)	1277 (0.51%)	53	1387 (0.55%)	11,715 (04.67%)	45,024 (17.93%)	76,275 (30.38%)	116,639 (46.46%)
2009	254,363	10,564 (4.15%)	60	80,582 (31.68%)	129,878 (51.06%)	97,527 (38.34%)	3764 (1.48%)	2453 (0.96%)	17,332 (6.81%)	1274 (0.50%)	54	1373 (0.54%)	11,425 (04.49%)	40,904 (16.08%)	78,664 (30.93%)	121,989 (47.96%)
2010	257,120	10,538 (4.10%)	61	81,376 (31.65%)	130,034 (50.57%)	99,748 (38.79%)	3736 (1.45%)	2493 (0.97%)	17,579 (6.84%)	1284 (0.50%)	54	1381 (0.54%)	11,309 (04.40%)	37,558 (14.61%)	79,937 (31.09%)	126,929 (49.37%)
2011	260,344	11,014 (4.23%)	63	82,178 (31.75%)	130,357 (50.07%)	102,181 (39.25%)	3715 (1.43%)	2518 (0.97%)	17,877 (6.87%)	1307 (0.50%)	55	1386 (0.53%)	11,251 (04.32%)	34,774 (13.36%)	80,377 (30.87%)	132,551 (50.91%)
2012	262,123	10,878 (4.15%)	63	82,373 (31.43%)	129,973 (49.58%)	103,961 (39.66%)	3685 (1.41%)	2531 (0.97%)	18,048 (6.89%)	1327 (0.51%)	55	1303 (0.50%)	10,921 (04.17%)	32,392 (12.36%)	79,065 (30.16%)	138,437 (52.81%)
2013	262,254	10,945 (4.17%)	64	82,070 (31.29%)	128,847 (49.13%)	105,027 (40.05%)	3640 (1.39%)	2520 (0.96%)	18,080 (6.89%)	1328 (0.51%)	56	1160 (0.44%)	10,299 (03.93%)	29,964 (11.43%)	76,837 (29.30%)	143,989 (54.91%)
2014	261,087	11,054 (4.23%)	64	81,495 (31.21%)	127,140 (48.70%)	105,558 (40.43%)	3591 (1.38%)	2472 (0.95%)	18,078 (6.92%)	1314 (0.50%)	57	978 (0.37%)	9555 (03.66%)	27,563 (10.56%)	73,700 (28.23%)	149,288 (57.18%)
2015	258,370	11,050 (4.28%)	65	80,400 (31.12%)	124,940 (48.36%)	105,099 (40.68%)	3511 (1.36%)	2465 (0.95%)	17,949 (6.95%)	1315 (0.51%)	58	781 (0.30%)	8829 (03.42%)	24,921 (09.65%)	69,433 (26.87%)	154,405 (59.76%)
2016	252,053	11,573 (4.59%)	66	78,232 (31.04%)	121,176 (48.08%)	102,974 (40.85%)	3417 (1.36%)	2424 (0.96%)	17,612 (6.99%)	1262 (0.50%)	58	540 (0.21%)	7829 (03.11%)	22,467 (08.91%)	63,606 (25.24%)	157,610 (62.53%)
CDC 2016^∗^	977,883	NA	NA	229,231 (23.40%)	300,483 (30.11%)	405,738 (40.66%)	41,647 (4.17%)	13,411 (1.34%)	211,956 (21.24%)	3775 (0.38%)	NA	28,485 (2.90%)	144,049 (14.7%)	188,789 (19.30%)	308,597 (31.60%)	307,956 (31.50%)

### 3.2 Association of comorbidities with HIV infection

We plotted MLE and 95% confidence for upper and lower bounds. MLE values above 0 are associated with HIV+ cases and MLE values below 0 are associated with HIV– controls. Out of the 66 comorbidities, 12 were relatively rare affecting <5000 (2% or fewer) HIV positive patients in any model year. Another 23 comorbidities had negative or neutral (not statistically significant) MLEs in most years, meaning that they were not associated with HIV infection. We focus our presentation here on the remaining 31 comorbidities with >5000 cases and positive association with HIV infection. These 31 comorbidities could be divided into 3 groups.

### 3.3 Comorbidities with persistent positive association with HIV infection

**Figure 1:**
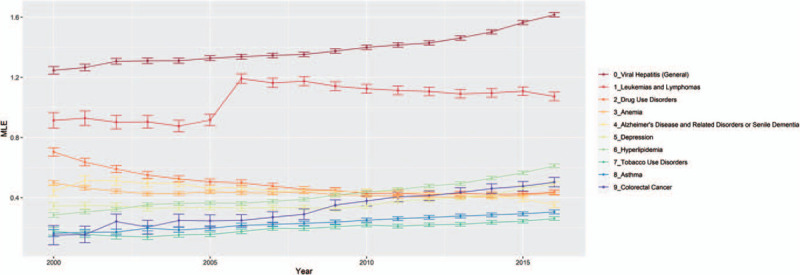
Comorbidities with persistent HIV+ association (higher MLE). The figure plots the higher range of maximum likelihood estimate (*y* axis) for each comorbidity that maintained HIV+ association (MLE above 0) across yearly (*x* axis) logistic regression models. Confidence intervals at 95% (error bars) are plotted along with the yearly model MLE by comorbidity type. Logistic regression models were computed using the proc logistic procedure in SAS 9.4. HIV = human immunodeficiency virus, MLE = maximum likelihood estimate.

**Figure 2:**
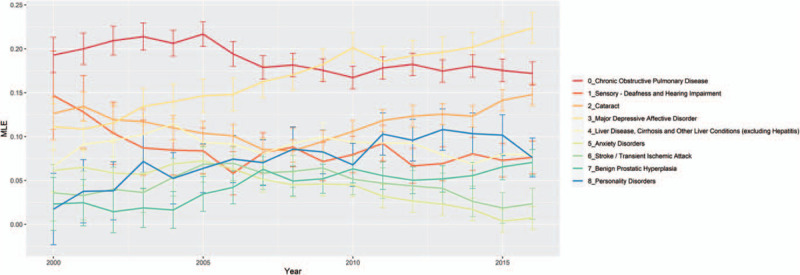
Comorbidities with persistent HIV+ association (higher MLE). The figure plots the lower range of maximum likelihood estimate (*y* axis) for each comorbidity that maintained HIV+ association (MLE above 0) across yearly (*x* axis) logistic regression models. Confidence intervals 95% (error bars) are plotted along with the yearly model MLE by comorbidity type. Logistic regression models were computed using the proc logistic procedure in SAS 9.4. HIV = human immunodeficiency virus, MLE = maximum likelihood estimate.

We identified 19 comorbidities with significantly positive HIV associations in at least half of the observation years (Figs. 1 and 2). They could broadly be separated into 2 subgroups, 10 high association morbidities (MLE always above 0.3, Fig. 1), and 9 low association morbidities (MLE always below 0.3 but above 0, Fig. 2). The high association morbidities were viral hepatitis, leukemias and lymphomas, hyperlipidemia, colorectal cancer, depression, drug use disorders, anemia, Alzheimer disease and related disorders, asthma, and tobacco use disorders. Low association morbidities included major depressive affective disorder, chronic obstructive pulmonary disease, cataract, liver disease (excluding viral hepatitis), deafness and hearing impairment, personality disorders, benign prostatic hyperplasia, stroke or transient ischemic attack, and anxiety disorders.

### 3.4 Comorbidities that became positively associated with HIV infection during the study period

**Figure 3:**
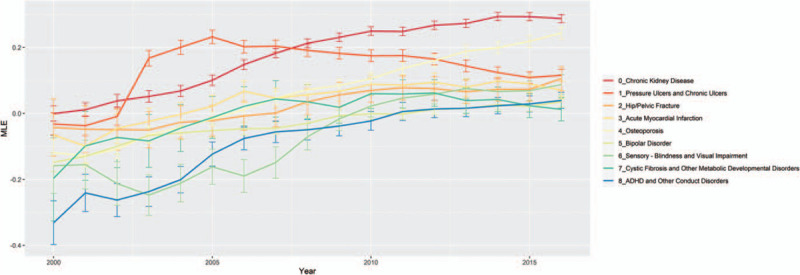
Comorbidities that change association to HIV+ cases. The figure plots the maximum likelihood estimate (*y* axis) for each comorbidity that changed from HIV– association (MLE below 0) to HIV+ (MLE above zero) association across yearly (*x* axis) logistic regression models. Confidence intervals 95% (error bars) are plotted along with the yearly model MLE by comorbidity type. Logistic regression models were computed using the proc logistic procedure in SAS 9.4. HIV = human immunodeficiency virus, MLE = maximum likelihood estimate.

Nine comorbidities acquired HIV positive association during our study period (Fig. 3). These included acute myocardial infarction, osteoporosis, hip/pelvic fracture, chronic kidney disease, bipolar disorder, pressure ulcers and chronic ulcers, cystic fibrosis and other metabolic developmental disorders, blindness and visual impairment, and ADHD (Attention Deficit Hyperactivity Disorder) and other conduct disorders. Among these conditions, hip/pelvic fracture, chronic kidney disease and pressure ulcers and chronic ulcers began with neutral (insignificant) association and flipped to positive association by the end of the study period, while the remaining conditions changed from negative to positive association.

### 3.5 Comorbidities that became less associated with HIV infection during the study period

**Figure 4:**
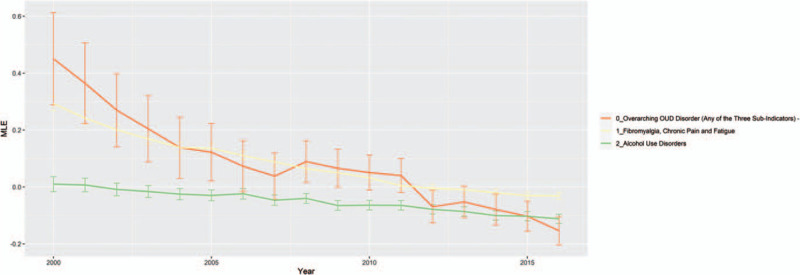
Comorbidities that became associated with HIV– controls. The figure plots the maximum likelihood estimate (*y* axis) for each comorbidity that changed from HIV+ association (MLE above zero) to HIV– (MLE below 0) association across yearly (*x* axis) logistic regression models. Confidence intervals 95% (error bars) are plotted along with the yearly model MLE by comorbidity type. Logistic regression models were computed using the proc logistic procedure in SAS 9.4. HIV = human immunodeficiency virus, MLE = maximum likelihood estimate.

Three comorbidities became less associated with HIV infection during our observation period: alcohol use disorder (AUD), opioid use disorder – overarching (OUD-O) as well as fibromyalgia, chronic pain, and fatigue (Fig. 4). AUD had neutral association in early years and became negatively associated with HIV infection in later years. OUD-O was a composite status that evaluated 3 other OUD comorbidities and returned true if any of the underlying OUD comorbidities were true. The 3 underling OUD comorbidities were OUD treatment in a hospital or emergency department, medication assisted treatment for OUD and OUD diagnosed on a claim with specific ICD-9-CM or ICD-10-CM codes. OUD-O was positively associated with HIV infection in early years but became negatively associated in later years. Fibromyalgia, chronic pain, and fatigue were positively associated in early years, but the association became neutral in later years.

## 4 Discussion

To the best of our knowledge, among similar studies of comorbidities in HIV infected patients, our study contains the biggest sample of patients in the US. According to CDC estimates, around 1.1 million people were living with HIV at the end of 2016, of which about 14% did not know they had HIV.^[21]^ Our study covers about a quarter (25.7%) of known HIV infected individuals in the US. We cover the 17-year period from 2000 to 2016, during which both the anti-HIV treatment options and outcomes had undergone significant evolution. Since we focus on prevailing trends occurring over many years, we are less liable to be distracted by chance findings of “significant” results in point-to-point cross-sectional studies. Compared with studies that modeled comorbidities individually, ours is a regression study using all comorbidities as covariates, which is more robust and can adjust for the influence of multiple comorbidities. Since our methodology relies on a stable and continuous source of Medicare claims data, the analysis can be repeated as necessary to observe changes over time. Moreover, the data set we use is publicly available, so our results can be independently validated.

There are 2 important caveats in interpreting our results. Firstly, the Medicare HIV population may not be representative of the overall US HIV population. Secondly, since the normal age for Medicare eligibility is 65 years, our study subjects (both cases and controls) under 65 years (making up 78% of our HIV positive cases), need to be additionally qualified to receive Medicare. The most common reason is through Social Security Disability Insurance (SSDI) that entitles a patient to Medicare coverage at any age if they present with some qualifying disability. For this reason, our controls should be considered to have more health issues compared with the general population. If a comorbidity is found to be positively associated with HIV infection over controls, the fact that the controls are sicker than the general population would lend more weight to the observation. On the other hand, if a comorbidity is negatively or neutrally associated with HIV infection, the result should be interpreted with care.

A case in point is the rather surprising finding in our study that opioid use disorder (OUD) has changed from positively associated with HIV to negatively associated, despite the known association of intravenous drug use and HIV transmission. One explanation is there has been a bigger increase in OUD among our controls compared with cases. Patients with disabling injuries and musculoskeletal problems make up a significant proportion of SSDI enrollees,^[22]^ and these patients are especially vulnerable to develop OUD. The interplay between HIV infection and OUD is complex and needs to be understood in the broader context of the opioid epidemic in the US, with its far-reaching medical-social consequences.^[37–40]^ Similar factors may be at play for the decreasing trend of association observed for fibromyalgia, chronic pain and fatigue, and alcohol use disorders.

The fact that our study reaffirms some morbidities that are well known to be more prevalent among HIV patients lends support to our method and results. Most of the conditions that are found to have persistently high positive association fall into this group. They include viral hepatitis, drug use disorders, anemia, hyperlipidemia, tobacco use disorder, depression, dementia, and some cancers. Perhaps more interesting is the finding that some conditions have acquired positive association in the course of our study period. This may reflect the impact of changing demographics of HIV patients or evolution in treatment. We highlight some conditions here for discussion.

### 4.1 Bone mineral density, osteoporosis, and fractures

Our observation period from 2000 to 2016 witnessed the emergence of what we would now consider popular HIV medications like: tenofovir-emtricitabine (Truvada), approved in 2004 and tenofovir-emtricitibine-efavirenz (Atripla), approved in 2006.^[23,24]^ Many HIV medications (especially protease inhibitors) have on-label warnings about bone mineral density, osteoporosis, and fracture.^[25–28]^ Our results did show that osteoporosis and fractures become more positively associated with HIV infection over the observation period. Another factor which may be at play is the increasing association of HIV infection with viral hepatitis. Some studies have shown that hepatitis-C could be a risk factor for osteoporosis and fracture.^[26,28]^

### 4.2 Chronic kidney disease

Chronic kidney disease (CKD) was observed as changing its HIV association from neutral to positive. This trend is particularly alarming because our controls already have a higher than normal incidence of CKD, which is one of the qualifying conditions for Medicare enrollment before the age of 65. Many emerging HIV medications over the study period are implicated in directly causing or exacerbating an existing kidney injury.^[29–32]^ Protease inhibitors and specific combination therapies have been implicated in CKD.^[28]^ Preventing CKD is key to the survival of these patients as HIV positive patients are difficult organ transplant candidates and dialysis cases.

### 4.3 Acute myocardial infarction

Several studies have documented concerns of disproportionate prevalence of acute myocardial infarction (AMI) among HIV positive patients.^[33–36]^ The higher incidence of AMI could be explained partly by the improved survival, as more HIV positive patients are living to older age, at which the natural incidence of AMI is higher. However, our age-controlled study still shows a HIV positive association that has developed over the past decade. The emergence of AMI as a HIV positive comorbidity is concerning. Some HIV medications or the infective process itself may contribute to AMI risk. In our study, the HIV associations for ischemic heart disease and heart failure were always negative, meaning that these conditions were associated with controls rather than HIV positive cases. This may suggest that AMI among HIV positive patients could have different pathophysiology compared with HIV negative patients.

### 4.4 Blindness and visual impairment

Before the introduction of highly active antiretroviral therapy (HAART), 50% to 75% of HIV infected patients developed visual problems.^[37]^ Cytomegalovirus retinitis was the most frequent cause of visual loss in patients with AIDS. With the widespread use of HAART, other causes of visual loss are becoming more important. Among them is immune recovery uveitis (IRU), which is the paradoxical worsening of treated opportunistic infection after the initiation of antiretroviral therapy.^[38]^ Immune recovery uveitis is thought to be due to a dysregulation of the expanding population of CD4+ T cells specific for a co-infecting opportunistic pathogen such as cytomegalovirus. IRU is now one of the leading causes of ocular morbidity in HIV patients. Some studies have found higher incidence of cataract among HIV positive patients, which may also be related to IRU.^[39,40]^ Ischemic maculopathy is another cause of ocular morbidity which has been found to be caused by HIV microvasculopathy. Other conditions that contribute to visual impairment in HIV patients include uveitis, glaucoma, Herpes Zoster ophthalmicus, optic nerve disease, and diabetic retinopathy.^[41–43]^

### 4.5 Skin ulcers

Increased incidence of pressure ulcers has been reported in HIV infected patients.^[44]^ Potential contributing factors include muscle weakness, emaciation, and loss of adipose tissue associated with chronic illness, which make HIV patients more susceptible for development of pressure ulcers.^[45]^ It has also been observed that there is an increased incidence of venous ulcers in HIV infected patients.^[46]^ One hypothesis is that this is related to venous insufficiency secondary to repeated self-injection in lower limb veins in intravenous drug users.

Apart from the above comorbidities, 3 other conditions have acquired positive HIV association during our study period. The rise of bipolar disorder could be related to the increased incidence and awareness of depressive illnesses in HIV infected patients. The other 2 comorbidity labels are rather heterogeneous. Cystic fibrosis and other metabolic developmental disorders include non-specific conditions like unspecified vitamin deficiency, which could be related to nutritional insufficiency in HIV patients. ADHD and other conduct disorders include diagnoses such as delinquency and disruptive behavior, which could be related to the psychosocial challenges of living with HIV infection. Sometimes HIV related dementia, which includes attention issues is coded as ADHD in U.S. billing systems due to the lack of a specific ICD10-CM code for “HIV related dementia,” not to be confused with the more severe yet similarly named “AIDS related dementia.”^[47,48]^

Our study has the following limitations. Our data did not allow us to estimate the date of first HIV diagnosis. For this reason, we have not been able to study the effect of the length of HIV infection on comorbidities. Our study did not cover HIV patients who received care outside of Medicare and Medicaid. One example is the Ryan White program run by the Health Resources and Services Administration. Our comorbidity data only came from Medicare data, so it is possible (though unlikely) that comorbidities only appearing on Medicaid claims would be missed.

## 5 Conclusion

Medicare claims are a useful source of data for HIV related research. The Medicare cohort covers a quarter of all US patients living with HIV infection. Through a case-control retrospective study, we managed to reaffirm some comorbidities (e.g., viral hepatitis, drug use disorders) that are already known to be more prevalent in HIV positive patients. We also identified emerging concerns (e.g., acute myocardial infarction, chronic kidney disease) that have become more positively associated with HIV infection through the 17-year observation period. These trends are likely to be related to changes in patient demographics and evolution in treatment.

## Acknowledgment

The findings and conclusions in this article are those of the authors and do not necessarily represent the official position of NLM, NIH, or the Department of Health and Human Services.

## Author contributions

**Conceptualization:** Nick Williams, Kin Wah Fung.

**Data curation:** Craig Mayer.

**Formal analysis:** Nick Williams.

**Funding acquisition:** Vojtech Huser.

**Investigation:** Nick Williams, Frank Rhame, Kin Wah Fung.

**Methodology:** Nick Williams.

**Project administration:** Vojtech Huser, Kin Wah Fung.

**Resources:** Vojtech Huser, Craig Mayer.

**Software:** Nick Williams.

**Supervision:** Vojtech Huser, Kin Wah Fung.

**Validation:** Nick Williams, Frank Rhame.

**Visualization:** Nick Williams.

**Writing – original draft:** Nick Williams, Kin Wah Fung.

**Writing – review & editing:** Nick Williams, Vojtech Huser, Frank Rhame, Craig Mayer, Kin Wah Fung.
